# Blood levels of glial fibrillary acidic protein for predicting clinical progression to Alzheimer’s disease in adults without dementia: a systematic review and meta-analysis protocol

**DOI:** 10.1186/s41512-024-00167-3

**Published:** 2024-03-05

**Authors:** Takashi Nihashi, Keita Sakurai, Takashi Kato, Yasuyuki Kimura, Kengo Ito, Akinori Nakamura, Teruhiko Terasawa

**Affiliations:** 1 Department of Radiology, National Center for Geriatrics and Gerontology, 7-430 Morioka-Cho, Obu, Aichi 474-8511 Japan; 2 Department of Biomarker Research, National Center for Geriatrics and Gerontology, 7-430 Morioka-cho, Obu, Aichi 474-8511 Japan; 3 Department of Clinical and Experimental Neuroimaging, National Center for Geriatrics and Gerontology, 7-430 Morioka-Cho, Obu, Aichi 474-8511 Japan; 4 National Center for Geriatrics and Gerontology, 7-430 Morioka-Cho, Obu, Aichi 474-8511 Japan; 5 Section of General Internal Medicine, Department of Emergency Medicine and General Internal Medicine, Fujita Health University School of Medicine, 1-98 Dengakugakubo, Kutsukake-Cho, Toyoake, Aichi 470-1192 Japan

**Keywords:** Alzheimer’s disease, Biomarker, Dementia, Meta-analysis, Mild cognitive impairment, Prediction, Prognostic factor

## Abstract

**Background:**

There is urgent clinical need to identify reliable prognostic biomarkers that predict the progression of dementia symptoms in individuals with early-phase Alzheimer’s disease (AD) especially given the research on and predicted applications of amyloid-beta (Aβ)-directed immunotherapies to remove Aβ from the brain. Cross-sectional studies have reported higher levels of cerebrospinal fluid and blood glial fibrillary acidic protein (GFAP) in individuals with AD-associated dementia than in cognitively unimpaired individuals. Further, recent longitudinal studies have assessed the prognostic potential of baseline blood GFAP levels as a predictor of future cognitive decline in cognitively unimpaired individuals and in those with mild cognitive impairment (MCI) due to AD. In this systematic review and meta-analysis, we propose analyzing longitudinal studies on blood GFAP levels to predict future cognitive decline.

**Methods:**

This study will include prospective and retrospective cohort studies that assessed blood GFAP levels as a prognostic factor and any prediction models that incorporated blood GFAP levels in cognitively unimpaired individuals or those with MCI. The primary outcome will be conversion to MCI or AD in cognitively unimpaired individuals or conversion to AD in individuals with MCI. Articles from PubMed and Embase will be extracted up to December 31, 2023, without language restrictions. An independent dual screening of abstracts and potentially eligible full-text reports will be conducted. Data will be dual-extracted using the CHeck list for critical appraisal, data extraction for systematic Reviews of prediction Modeling Studies (CHARMS)-prognostic factor, and CHARMS checklists, and we will dual-rate the risk of bias and applicability using the Quality In Prognosis Studies and Prediction Study Risk-of-Bias Assessment tools. We will qualitatively synthesize the study data, participants, index biomarkers, predictive model characteristics, and clinical outcomes. If appropriate, random-effects meta-analyses will be performed to obtain summary estimates. Finally, we will assess the body of evidence using the Grading of Recommendation, Assessment, Development, and Evaluation Approach.

**Discussion:**

This systematic review and meta-analysis will comprehensively evaluate and synthesize existing evidence on blood GFAP levels for prognosticating presymptomatic individuals and those with MCI to help advance risk-stratified treatment strategies for early-phase AD.

**Trial registration:**

PROSPERO CRD42023481200.

**Supplementary Information:**

The online version contains supplementary material available at 10.1186/s41512-024-00167-3.

## Background

Alzheimer’s disease (AD) is the most common type of dementia, accounting for approximately 60–80% of all dementia cases [1, 2]. In 2019, the “Global Burden of Diseases, Injuries, and Risk Factors Study” reported that an estimated 57 million people worldwide were living with dementia, and this number is projected to increase to more than 150 million by 2050 [3]. Although the pathogenesis of AD is not fully elucidated, the accumulation of amyloid β (Aβ) peptides coupled with the spread of tau pathology is believed to play an integral role in disease progression [1, 2]. The current diagnostic criteria necessitate confirmation of brain accumulation of both Aβ and tau through positron emission tomography (PET) or central spinal fluid (CSF) testing [4].

Recent advances have added newly developed immunotherapies for Aβ to the conventional standard of care treatment which include cholinesterase inhibitors and memantine, an N-methyl-D-aspartate receptor antagonist. The current clinically available immunotherapy drugs for Aβ include aducanumab, an Aβ fibril-directed antibody (approved in the USA only), and lecanemab, an Aβ protofibril-directed antibody (approved in the USA and Japan), and while they have proven to reduce Aβ in the brain, only lecanemab has been demonstrated to slow cognitive and functional decline in patients with mild cognitive impairment (MCI) or mild dementia due to AD [5, 6]. However, Aβ-directed immunotherapies are costly and can cause potentially serious and non-negligible adverse effects, including amyloid-related cerebral hemorrhage and edema in addition to infusion-related reactions [7]. Although accumulation of amyloid and tau in the brain is commonly observed in older populations, a significant proportion of individuals with normal cognitive function exhibit these biomarkers and do not progress to AD [8]. Therefore, it is necessary to predict cognitive and functional decline and to support treatment decisions and accurate prediction of the risk of future progression to AD among pre- or early symptomatic, biomarker-positive individuals. Moreover, it is imperative to identify the groups for whom these test and treatment strategies should be pursued.

Glial fibrillary acidic protein (GFAP) is a high-profile astrocyte-derived biomarker that reflects astrocyte activation and neuroinflammation [9]. Several imaging, CSF, and blood biomarker studies have demonstrated that GFAP levels are higher in patients with MCI and/or AD than in cognitively unimpaired individuals, suggesting the potential for GFAP to complement existing AD biomarkers as a possible indicator of the neuroinflammatory aspects of neurodegenerative progression [10–12]. Additionally, a longitudinal biomarker study assessing the association between GFAP and phosphorylated tau levels in the plasma also proposed astrocyte reactivity as an upstream event, linking Aβ with initial tau pathology [13]. This has led to the proposal of adding neuroinflammatory biomarkers as “I” to the conventional classification system based on amyloid, tau, and neurodegeneration to describe the progression of AD (amyloid, tau, neurodegeneration (ATN) classification) [14] to the proposed ATN(I) classification [15].

Based on the studies discussed above and recently proposed expert recommendations on the appropriate use of blood biomarkers, including phosphorylated tau (p-tau), in clinical practice [16, 17], this systematic review has two specific objectives focused on GFAP as an important upstream factor during AD development. The first objective is to summarize the prognostic value of blood GFAP levels in predicting the future progression of cognitive decline in cognitively unimpaired individuals or those with MCI. The second objective is to identify and summarize all the prediction models that incorporated blood GFAP levels as a predictor of the future progression of cognitive impairment in the same two target populations.

## Methods

This systematic review and meta-analysis protocol will follow the Preferred Reporting Items for the Systematic Review and Meta-Analysis Protocols 2015 statement [18]. In the event of protocol amendments, the date of each amendment will be accompanied by a description of the change and rationale.

Two key questions (KQs) were developed based on the PROGnosis REsearch Strategy (PROGRESS) framework for prognostic research [19–22].

- KQ1 (PROGRESS type II, prognostic factor research [20]): Can blood GFAP levels predict the risk of progression of cognitive dysfunction in cognitively unimpaired individuals or in those with MCI?
- KQ2 (PROGRESS type III, prognostic model research [21]): Can risk assessment models (RAMs) incorporating blood GFAP levels predict the future risk of cognitive dysfunction progression in cognitively unimpaired individuals or those with MCI?

### Information sources and search strategies

We will search the PubMed and Embase databases from database inception through December 31, 2023, using both subject indexing (i.e., MeSH and Emtree, respectively) and free-text terms. The covered terms will include “preclinical dementia,” “mild cognitive impairment,” “Alzheimer disease,” and “glial fibrillary acidic protein,” as well as their synonyms. The complete search strategy and list of databases are available in the Supplementary file (Appendix 1). The electronic search results will be imported into EndNote 21 (Clarivate Analytics, Philadelphia, USA), and duplicate results will be removed. For additional searches, we will screen the reference lists of eligible studies as well as the reference lists of systematic reviews and meta-analyses related to this topic, which will be identified through this database search. No language restrictions will be imposed.

### Eligibility criteria

Table 1 presents the detailed inclusion criteria based on the populations, interventions, comparator interventions, outcomes, timings, and setting frameworks modified for prognostic factor reviews [23]. We will include any prospective or retrospective cohort study that includes at least 15 adult individuals in whom baseline blood GFAP levels and the progression of cognitive impairment were assessed at least 1 year after baseline. We will target two specific adult populations of clinical interest: (i) individuals aged 50 years or older without cognitive impairment and (ii) individuals aged 50 years or older with MCI [24, 25], regardless of modifiable risk factors (e.g., diabetes, hypertension, obesity, smoking, high alcohol consumption, sedentary lifestyle, and low educational attainment). In addition, we will target a third population of clinical interest: (iii) high-risk individuals aged ≥ 18 years. Here, “high risk” for developing dementia is defined as individuals with ≥ 1 unmodifiable risk factor, such as genetic variants (e.g., apolipoprotein ε4 [APOE4] copy numbers) or chromosomal abnormalities (e.g., Down syndrome).

**Table 1:** Inclusion criteria based on the PICOTS framework modified for prognostic factor and model studies

PICOTS	Specific details
*P*opulation	(i) Adults (aged ≥ 50 years) without cognitive impairment at baseline
	(ii) Adults (aged ≥ 50 years) with MCI at baseline
	(iii) High-risk individuals aged ≥ 18 years. Here, “high risk” for developing dementia is defined as individuals with ≥ 1 unmodifiable risk factor, such as genetic variants (e.g., APOE4 copy numbers) or chromosomal abnormalities (e.g., Down syndrome)
*I*ndex prognostic factor (KQ1)	Blood GFAP levels sampled and assessed at baseline
*C*omparator prognostic factors (KQ1)	Age, sex, APOE4, Aβ, and other biomarkers including p-tau
*I*ndex and *c*omparator RAMs (KQ2)	Any RAMs incorporating blood GFAP levels and other prognostic factors
*O*utcomes	Clinical conversion of cognitive impairment as the binary outcomes
	(i) From no cognitive impairment to MCI or AD
	(ii) From MCI to AD
*T*iming	At least 1 year from baseline
*S*etting	Blood sampling (for GFAP level measurement) performed in both primary and secondary care

*Abbreviations*: *Aβ* Amyloid β, *AD* Alzheimer disease, *APOE4* Apolipoprotein ε4, *GFAP* Glial fibrillary acidic protein, *KQ* Key question, *MCI* Mild cognitive impairment, *PICOTS* Populations, interventions, comparator interventions, outcomes, timings, and settings, *p-tau* Phosphorylated tau, *RAM* Risk assessment model

Two independent reviewers will double-screen the abstracts using Abstrackr, a web-based software for citation screening (Center for Evidence Synthesis in Health, Brown University, Province, USA) [26]. All potentially eligible full-text articles selected by at least one reviewer will pass the screening process. All non-English publications will be translated into English using Google Translate (Google, Mountain View, CA, USA) before full-text evaluation. We will then review the full texts for eligibility. Any discrepancies in the full-text assessment will be resolved via consensus.

### Data extraction

Two reviewers will extract data items recommended by the CHeck list for critical Appraisal, data extraction for systematic Reviews of prediction Modeling Studies (CHARMS) [27] and the CHARMS prognostic factor (PF) checklist [28]. The developed and piloted extraction form is available in the Supplementary file (Appendix 2). One primary reviewer will extract descriptive data, and another will verify all the extracted data. Two independent reviewers will double-extract quantitative data for outcomes of interest. A consensus will be used to resolve discrepancies. We will contact the study authors via email for missing or unresolved quantitative data. We will send two additional email correspondences if no response is received 2 weeks after the previous correspondence attempt.

### Source data characteristics

We will include the study identification (first author and publication year), study design (cohort, case–control, randomized trial, or registry data), and cohort or registry names.

### Participant characteristics

We will include the eligibility criteria (inclusion and exclusion criteria), enrollment methods (consecutive or random vs. non-consecutive), study location (country and city), number of centers, clinical setting, participant descriptions including average age (mean with standard deviation and/or median and interquartile or minimum–maximum range), percentage of male sex, and enrollment date.

### Predictor characteristics

We will include the number, type, definition, measurement time, and methods of any predictors used in (i) multivariable models that examined how well GFAP could predict the outcome of interest as an independent variable (KQ1) or (ii) reported RAMs that included GFAP as a predictor and predicted the outcome of interest (KQ2). We will also examine whether the predictors were assessed blinded to the outcome of interest and how the data type of the results was handled or transformed in statistical modeling. Regarding the measurement of blood GFAP levels, we will include the source of blood (whole blood, plasma, or serum), timing of measurement after blood drawing, storage-related parameters (temperature and duration), types of assays and platforms, and cutoff values if specified. For additional candidate predictor characteristics, we will include patient characteristics (demographics, participant history, and physical examination, including cognitive function tests and comorbidities) and biomarkers employed in the GFAP-incorporated RAMs. For any blood or CSF biomarker other than GFAP, we will extract information on the sample sources, timing of the measurement after sample collection, storage-related parameters (temperature and duration), and types of assays and platforms used. For imaging biomarkers using PET, the technical specifications and interpretation methods regarding the PET will be extracted.

### Outcome characteristics

The primary outcome of interest will be clinical conversion to cognitive impairment (binary outcome). We will define clinical conversion as (i) conversion to MCI or AD in cognitively unimpaired individuals and (ii) conversion to AD in individuals with MCI. We will use the Petersen criteria for the definition of MCI [24]. AD will be defined according to the criteria from the National Institute of Neurological and Communicative Disorders and Stroke and the Alzheimer’s Disease and Related Disorders Association [29] or the National Institute on Aging-Alzheimer’s Association [30]. We will additionally retrieve information on the timing of outcome events and/or overall follow-up duration after baseline, when predictors were evaluated. We will also ascertain whether the assessment of outcomes was conducted in a blinded manner with respect to the predictors. In cases where a study analyzed AD alongside other forms of dementia and the data specific to AD alone cannot be independently extracted, the information will be utilized in the sensitivity analysis.

### Sample size and missing data

We will extract the number of participants, outcome events, and events per candidate predictor (i.e., the number of outcome events divided by the number of predictive variables) employed in the multivariate models (to assess GFAP’s prognostic ability of GFAP; KQ1) or GFAP-incorporated RAMs (KQ2). For missing predictors, we will extract the number of participants with any missing values and the number of participants with missing data for each predictor. In addition, we will record how the missing data were handled. We will assess attrition (i.e., loss to follow-up) by extracting the number of censored observations in each category for GFAP levels or other categorical PFs.

### Model development and evaluation

We will extract the modeling method, the method for selecting predictors for inclusion in multivariate modeling, and the criteria used. Any evidence of nonproportional hazards of blood GFAP and other predictors, if reported, will be recorded. We will also record whether the shrinkage of the predictor weights or regression coefficients was adjusted for overfitting (KQ2 only). For model evaluation, we will extract how the model performance was tested (e.g., development dataset only or separate external validation) and whether the model was adjusted or updated (KQ2 only).

### Quantitative data

We will extract point estimates and their variances or 95% confidence intervals (CIs). We will also extract the unadjusted and adjusted hazard ratios (HRs) for blood GFAP levels, with the set of adjustment factors used for the adjusted estimates (KQ1). When relevant data is not directly extractable, we will use the standard approach [31, 32] or the ratio of the logarithms of event-free proportions [33] to obtain the unadjusted HR estimates and their variances. To standardize the sets of adjustment factors, we will predefine the following hierarchy based on the minimum set of adjustment factors [28].

- Level 0: Unadjusted (no covariates)
- Level 1: Age and sex
- Level 2: Age, sex, and APOE4
- Level 3: Age, sex, and amyloid (± APOE4)
- Level 4: Age, sex, amyloid (± APOE4), and other biomarkers including p-tau

For GFAP-incorporated RAMs, we will extract the calibration slope, calibration-in-the-large (CITL), and expected/observed or observed/expected outcome event ratios (E/O or O/E, respectively) as measures of calibration. Additionally, we will gather information on the C-statistic and D-statistics as measures of discrimination (KQ2 only).

### Risk-of-bias assessment

Two independent reviewers will assess the risk of bias in the primary studies using established risk-of-bias assessment tools, the Quality In Prognosis Studies (QUIPS) [34], and the Prediction Study Risk-of-Bias Assessment Tool (PROBAST) [35], and any discrepant results will be resolved by consensus.

For studies that assessed the prognostic ability of blood GFAP levels as an independent PF (KQ1), we will use the QUIPS [34]. We will first assess the signaling items as yes, no, or no information and then rate the risk of bias as high, moderate, or low for (i) study participation, (ii) study attrition, (iii) PF measurement, (iv) outcome measurement, (v) adjustment for other factors, and (vi) statistical analysis and reporting.

For studies that assessed GFAP-incorporated RAMs (KQ2), we will use the PROBAST [35]. First, we will address the signaling questions as yes, no, or no information and then rate the risk of bias and concerns about applicability (except for analysis) as high, low, or unclear for (i) participation, (ii) predictors, (iii) outcomes, and (iv) analyses.

### Data synthesis

For studies involving blood GFAP levels as an independent PF (KQ1), the results will be sorted by (i) whether they were unadjusted or adjusted (based on the hierarchical levels described above), (ii) sample collection and measurement methods, and (iii) cutoff points if analyzed as binary or ordinary variables. Results will then be visually assessed using forest plots. If a group of results is reasonably similar after sorting the abovementioned three attributes, we will perform a meta-analysis.

For studies pertaining to GFAP-incorporated RAMs (KQ2), this systematic review will encompass models at various stages, including development and validation studies. Consequently, we expect significant clinical heterogeneity across studies exploring the use of blood GFAP levels as a predictive factor. Therefore, we will first sort the results by the sets of included predictors and descriptively examine the similarities and discrepancies using graphs and tables. We will perform a meta-analysis only if a reasonable number of studies validating an identical RAM are available.

If the data are amenable to quantitative synthesis, we will perform an aggregate study-level random-effects meta-analysis using the standard approximate normal-normal model based on the restricted maximum likelihood estimator [23]. For calibration slopes, CITLs, and D-statistics, the summary estimates will be combined in the original scale; E/Os or O/Es and HRs will be transformed to a log scale, and C-statistics will be transformed to a logit scale before performing the meta-analysis [23]. We will estimate corrected 95% CIs and prediction intervals (PIs) based on the Hartung-Knapp-Sidik-Jonkman method [36] and the Higgins-Thompson-Spiegelhalter method [37], respectively. Statistical heterogeneity will be visually assessed using forest plots and quantitatively using the random-effect standard deviation parameter, tau, and PIs of the effect size [38].

We will analyze the data of cognitively unimpaired populations and populations with MCI separately. In the main analysis, we will jointly analyze populations with subjective cognitive decline [39] alongside cognitively unimpaired populations. In the sensitivity analysis, these two categories will be analyzed separately as subgroups.

### Additional analyses

To assess small-study effects and publication biases, we will use funnel plot asymmetry only when at least 10 studies assess similar results [40]. To assess certainty in the body of evidence, we will use the Grading of Recommendations Assessment, Development, and Evaluation approach designed for prognostic studies [41, 42] and rate each outcome based on the validity, inconsistency, imprecision, and indirectness of each study.

### Statistical software

All statistical analyses will be performed using the Stata V.18.0/SE software (Stata Corp., College Station, TX, USA). All tests will be two-sided, and statistical significance will be defined as *p* < 0.05.

### Ethics and dissemination

An ethics review is not necessary, as this is a systematic review of publicly available data. The reviewed findings will be reported according to the transparent reporting of multivariable prediction models for individual prognosis or diagnosis checklist for systematic reviews and meta-analyses guidelines [43] and disseminated through publications in peer-reviewed journals.

## Discussion

### Interpretation

GFAP is a promising biomarker for predicting cognitive impairment in individuals with early AD [16, 17]. With multiple treatment options expected to become available in the near future, precise and efficient identification of progressor groups for improved treatment selection during early disease phases will be of paramount importance. When multiple costly therapeutic interventions are actively administered to a large number of individuals, there is a pressing need for an objective and rigorous evaluation of the efficacy of these therapies. However, cognitive function inevitably declines with age, regardless of AD pathology; thus, effectively differentiating pathological cognitive decline from physiological decline due to aging is not straightforward and may be further complicated by environmental factors such as patients’ lifestyles, available support networks, and preexisting diseases [8]. In this complex and diverse context, reliable biomarkers that can assist in the accurate and objective diagnosis and predict cognitive decline and other intermediate outcomes are urgently needed.

Efforts to establish clinical evidence for these blood biomarkers are currently underway, primarily in the research context with their application in clinical practice expected in the near future. Compared to PET and CSF testing, blood biomarkers are less invasive, easier to sample, and less costly; therefore, they potentially provide a reasonable and realistic option for repeated longitudinal evaluation and should be widely applied. Thus, our intention to perform a systematic review that rigorously assesses and summarizes emerging evidence on the prognostic ability of blood GFAP levels is timely and fruitful.

### Strengths and limitations of this study

A strength of this study is that it is the first formally planned systematic review and meta-analysis to comprehensively evaluate the existing evidence on the blood biomarker GFAP based on longitudinal cohort studies for prognosticating presymptomatic individuals and those with early AD. Existing systematic reviews have only focused on cross-sectional studies that compared blood GFAP levels between patients with established MCI and/or AD and presymptomatic cognitively unimpaired individuals [10–12]. This type of study, with a cross-sectional design, is easy to conduct and is an important source of data for testing the hypothesis that biomarker levels are associated with disease progression. However, this association must be validated through longitudinal studies. Another strength is that we will utilize a comprehensive literature search and the recommended up-to-date systematic review methodologies on prognostic factors and prognostic model studies to clarify the strengths and limitations of the current evidence [23].

An important limitation of this study is the heterogeneous design adopted in the primary prognostic or prediction model studies. Meta-analyses may not be feasible if similar types of data on the three attributes of a prognostic factor (i.e., covariates used for statistical adjustment, sample collection and measurement methods, and cutoff values) or data on identical RAMs are sparse.

In conclusion, this systematic review and meta-analysis will comprehensively evaluate and synthesize the existing evidence on blood GFAP levels for prognosticating presymptomatic individuals and those with MCI to help advance risk-stratified treatment strategies for early-phase AD.

### Supplementary Information

**Additional file 1: Appendix 1.** Search strategies. **Appendix 2.** Data extraction form.

## Data Availability

All data generated or analyzed during this study are included in this published article and its supplementary information files.

## Abbreviations

AD: Alzheimer’s disease
Aβ: Amyloid β
APOE4: Apolipoprotein ε4
ATN: Amyloid, tau, and neurodegeneration
CHARMS: CHeck list for critical appraisal and data extraction for systematic Reviews of prediction Modeling Studies
CI: Confidence interval
CITL: Calibration in the large
CSF: Cerebral spinal fluid
E/O: Expected/observed outcome event ratio
GFAP: Glial fibrillary acidic protein
HR: Hazard ratio
KQ: Key question
MCI: Mild cognitive impairment
O/E: Observed/expected outcome event ratio
PET: Positron emission tomography
PF: Prognostic factor
PI: Prediction interval
PICOTS: Populations, interventions, comparator interventions, outcomes, timings, and settings
PROBAST: Prediction Study Risk-of-Bias Assessment Tool
PROGRESS: Prognosis research strategy
QUIPS: Quality In Prognosis Studies
RAM: Risk assessment model
