# Supplementary material for: Blood levels of glial fibrillary acidic protein for predicting clinical progression to Alzheimer’s disease in adults without dementia: a systematic review and meta-analysis protocol
Source: Diagn Progn Res. 2024 Mar 5;8:4. doi: 10.1186/s41512-024-00167-3 (PMC10913586; doi:10.1186/s41512-024-00167-3)
Supplement: Supplementary file 1 — Additional file 1: Appendix 1. Search strategies. Appendix 2. Data extraction form. [file 41512_2024_167_MOESM1_ESM.docx]

**Online-Only Supplements**

## **Appendix 1. Search strategies**

***PubMed***

#1 "preclinical"[All Fields] OR "preclinically"[All Fields]

#2 "dementia"[MeSH Terms] OR "dementia"[All Fields] OR "dementias"[All Fields] OR "dementia s"[All Fields]

#3 #1 AND #2

#4 "MCI"[All Fields]

#5 "cognitive dysfunction"[MeSH Terms]

#6 "cognitive"[All Fields] AND "dysfunction"[All Fields]

#7 "cognitive dysfunction"[All Fields]

#8 "mild"[All Fields] AND "cognitive"[All Fields] AND "impairment"[All Fields]

#9 "mild cognitive impairment"[All Fields])

#10 "alzheimer disease"[MeSH Terms]

#11 "alzheimer"[All Fields] AND "disease"[All Fields]

#12 "alzheimer disease"[All Fields]

#13 "alzheimer"[All Fields]

#14 "alzheimer disease"[MeSH Terms]

#15 "alzheimer"[All Fields] AND "disease"[All Fields]

#16 #3 OR #4 OR #5 OR #6 OR #7 OR #8 OR #9 OR #10 OR #11 OR #12 OR #13 OR #14 #15

#17 "GFAP"[All Fields]

#18 "GFA"[All Fields]

#19 "GLIAL FIBRILLARY ACIDIC PROTEIN"[All Fields]

#20 #17 OR #18 OR #19

#21 "biological"[All Fields] AND "markers"[All Fields]

#22 "biomarker"[All Fields]

#23 "plasma"[MeSH Terms] OR "plasma"[All Fields]

#24 "serum"[MeSH Terms] OR "serum"[All Fields]

#25 #21 OR #22 OR #23 OR #24

#26 #20 AND #25

#27 #16 AND #26

***Embase (Elsevier)***

#1. preclinical AND ('dementia'/exp OR dementia)

#2. 'mild cognitive impairment' OR mci

#3. cognitive AND dysfunction

#4. 'alzheimer disease'

#5. alzheimer AND disease

#6. 'glial fibrillary acidic protein'

#7. gfap OR gfa

#8. 'biological marker'

#9. biomarker

#10. 'plasma'

#11. 'serum'

#12. #1 OR #2 OR #3 OR #4 OR #5

#13. #6 OR #7

#14. #8 OR #9 OR #10 OR #11

#15. #12 AND #13 AND #14

## **Appendix 2. Data extraction form**

***Source of data, participant, and outcome characteristics***

**CHARMS/CHARMS-PF**

| Study ID | Cohort or trial name | Design | Recruitment method | Age, yrs | Prevention of cognitive decline | Enrollment | Outcome | Dx criteria | Single vs. combined outcomes | Blinding assessment | Incorporation bias in dx | Time at outcome assessment | Average follow-up, yrs |
| --- | --- | --- | --- | --- | --- | --- | --- | --- | --- | --- | --- | --- | --- |
|  |  |  |  |  |  |  |  |  |  |  |  |  |  |
|  |  |  |  |  |  |  |  |  |  |  |  |  |  |

CHARMS = critical appraisal and data extraction for systematic reviews of prediction modelling studies; Dx = diagnosis; PF = prognostic factor

***Prognostic factor characteristics***

**CHARMS/CHARMS-PF**

| Study ID | Sample collection | Measurement from sampling, yrs | GFAP | Age | Sex | p-tau181, 217, 231, t-tau | Nfl | Aβ1-40, Aβ1-42 | *APOE* genotype | Others |
| --- | --- | --- | --- | --- | --- | --- | --- | --- | --- | --- |
|  |  |  |  |  |  |  |  |  |  |  |
|  |  |  |  |  |  |  |  |  |  |  |

Aβ = amyloid beta; APOE = apolipoprotein ε4; CHARMS = critical appraisal and data extraction for systematic reviews of prediction modelling studies; GFAP = glial fibrillary acidic protein; PF = prognostic factor

***Sample-size, missing data, and analysis characteristics***

**CHARMS-PF**

| Study ID | Sample size calculation | Events per participants | Events per variable | Participants with any MD | GFAP (MD handling) | Age (MD handling) | Sex (MD handling) | p-tau181, 217, t-tau (MD handling) | Nfl (MD handling) | *APOE* genotype (MD handling) | Others (MD handling) | Loss to follow-up or censored observations | Modeling method | Modeling assumption check or assessing non-proportional hazard | Prognostic factor selection in multivariable modeling | Selection or exclusion method for prognostic factors | Handling continuous prognostic factors |
| --- | --- | --- | --- | --- | --- | --- | --- | --- | --- | --- | --- | --- | --- | --- | --- | --- | --- |
|  |  |  |  |  |  |  |  |  |  |  |  |  |  |  |  |  |  |
|  |  |  |  |  |  |  |  |  |  |  |  |  |  |  |  |  |  |

APOE = apolipoprotein ε4; CHARMS = critical appraisal and data extraction for systematic reviews of prediction modelling studies; GFAP = glial fibrillary acidic protein; MD = missing data; Nfl = neurofilament light chain; PF = prognostic factor

**CHARMS**

| Study ID | Sample size calculation | Events per participants | Events per variable | Participants with any MD | GFAP (MD handling) | Age (MD handling) | Sex (MD handling) | Tau (MD handling) | Nfl (MD handling) | *APOE* genotype (MD handling) | Others (MD handling) | Modeling method | Modeling assumption check or assessing non-proportional hazard | Prognostic factor selection in multivariable modeling | Shrinkage method | Classification measures | Model validation | Model adjustment or update |
| --- | --- | --- | --- | --- | --- | --- | --- | --- | --- | --- | --- | --- | --- | --- | --- | --- | --- | --- |
|  |  |  |  |  |  |  |  |  |  |  |  |  |  |  |  |  |  |  |
|  |  |  |  |  |  |  |  |  |  |  |  |  |  |  |  |  |  |  |

APOE = apolipoprotein ε4; CHARMS = critical appraisal and data extraction for systematic reviews of prediction modelling studies; GFAP = glial fibrillary acidic protein; MD = missing data; Nfl = neurofilament light chain
